# A-Band Absorption Spectrum of the ClSO Radical: Electronic Structure of the Sulfinyl Group

**DOI:** 10.1021/acs.jpca.3c04977

**Published:** 2023-09-29

**Authors:** Wen Chao, Gregory H. Jones, Mitchio Okumura, Carl J. Percival, Frank A. F. Winiberg

**Affiliations:** † Division of Chemistry and Chemical Engineering, California Institute of Technology, 1200 E California Blvd, Pasadena, California 91125, United States; ‡ Jet Propulsion Laboratory, California Institute of Technology, 4800 Oak Grove Drive, Pasadena, California 91109-8099, United States

## Abstract

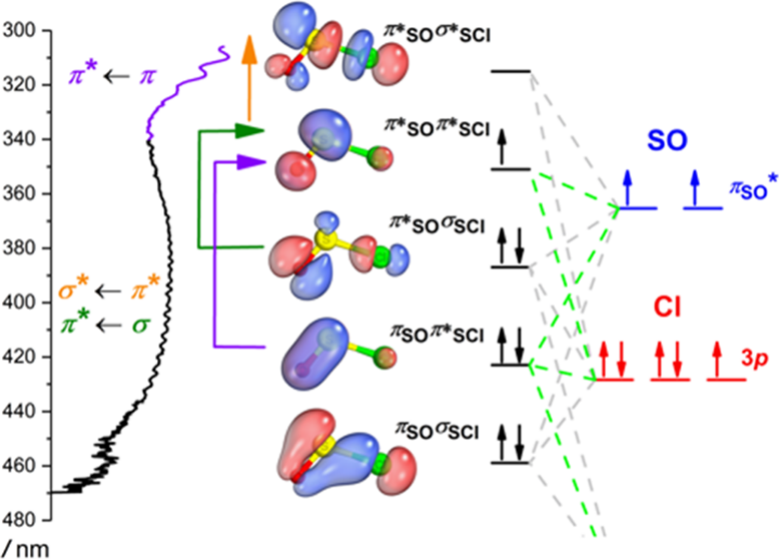

Sulfur oxide species (RSO_x_) play a critical role in many fields, ranging from biology to atmospheric chemistry. Chlorine-containing sulfur oxides may play a key role in sulfate aerosol formation in Venus’ cloud layer by catalyzing the oxidation of SO to SO_2_ via sulfinyl radicals (RSO). We present results from the gas-phase UV–vis transient absorption spectroscopy study of the simplest sulfinyl radical, ClSO, generated from the pulsed-laser photolysis of thionyl chloride at 248 nm (at 40 Torr of N_2_ and 292 K). A weak absorption spectrum from 350 to 480 nm with a peak at 385 nm was observed, with partially resolved vibronic bands (spacing = 226 cm^–1^), and a peak cross section σ(385 nm) = (7.6 ± 1.9) × 10^–20^ cm^2^. From *ab initio* calculations at the EOMEE-CCSD/ano-pVQZ level, we assigned this band to 1^2^A′ ← X^2^A″ and 2^2^A′ ← X^2^A″ transitions. The spectrum was modeled as a sum of a bound-to-free transition to the 1^2^A′ state and a bound-to-bound transition to the 2^2^A′ state with similar oscillator strengths; the prediction agreed well with the observed spectrum. We attributed the vibronic structure to a progression in the bending vibration of the 2^2^A′ state. Further calculations at the XDW-CASPT2 level predicted a conical intersection between the excited 1^2^A′ and 2^2^A′ potential energy surfaces near the Franck–Condon region. The geometry of the minimum-energy conical intersection was similar to that of the ground-state geometry. The lack of structure at shorter wavelengths could be evidence of a short excited-state lifetime arising from strong vibronic coupling. From simplified molecular orbital analysis, we attributed the ClSO spectrum to transitions involving the out-of-plane π/π^*^ orbitals along the S–O bond and the in-plane orbital possessing a σ/σ^*^ character along the S–Cl bond. We hypothesize that these orbitals are common to other sulfinyl radicals, RSO, which would share a combination of a strong and a weak transition in the UV (near 300 nm) and visible (400–600 nm) regions.

## Introduction

The formation of sulfur oxide species (RSO_x_), generated through reactions between oxygen and sulfinyl radicals (RSO), is important in many fields, including biology,^1,2^ organic synthesis,^3,4^ and atmospheric chemistry.^5^ For instance, an important source of sulfate aerosols in the Earth’s atmosphere is believed to be regulated by RSO_x_ oxidation, with HSO^6^ and SO_2_^7,8^ as intermediates in the formation of sulfates.^9^ However, in the middle atmosphere of Venus, condensed sulfuric acid clouds have been observed^10^ despite extremely low oxygen levels. Sulfate aerosol formation is attributed to the catalytic role of chlorine, involving in the oxidation of CO and SO to form CO_2_ and SO_2_.^11^ To comprehend the mechanism of chlorine catalysis of sulfinyl radical oxidation, direct kinetic measurements of sulfur species and chlorine in the laboratory must be conducted along with theoretical calculations to provide chemical insights.

The sulfinyl chloride radical, ClSO, is the smallest sulfinyl radical containing a chlorine atom and represents an ideal system for high-level *ab initio* calculations.^12^ Although the ClSO radical can be easily generated through the photolysis of thionyl chloride (Cl_2_SO),^13,14^ available spectroscopic measurements are limited to ground-state properties such as infrared (IR),^15^ far-IR,^16^ and electron paramagnetic resonance^17^ spectra. Furthermore, relevant theoretical studies are scarce.^18^ In the absence of suitable detection methods, kinetic investigations were limited to mass spectrometric detection.^19^

Recently, we observed a strong ultraviolet absorption spectrum of the ClSO radical in the gas phase near 300 nm, which we assigned to the 1^2^A″ ← X^2^A″ transition of the ClSO radical. We used the strong UV absorption to monitor ClSO and study the kinetics of the ClSO + Cl → Cl_2_SO reaction at pressures ranging from 10 to 90 Torr at 292 K.^12^

Previous studies in cryogenic matrices^20−24^ have shown that a few sulfinyl radicals exhibit both strong UV absorption near 300 nm and weak features near 400 nm, indicating that analogous weak features might also exist for ClSO. The goal of this work was to search for a longer-wavelength spectrum of gas-phase ClSO by transient absorption spectroscopy. We employed the same apparatus used to detect the shorter wavelength spectrum, a White-cell-based transient UV–vis absorption spectrometer coupled into a flow reactor.^12^ Radicals were generated by pulsed-laser photolysis, and transient absorption spectra were recorded in the 300–580 nm window. Low-lying excited electronic states were computed using Coupled Cluster and Complete Active Space Perturbation Theory (CASPT2) methods. The observed spectra were then assigned by comparison with the results from the *ab initio* calculations. Additionally, a molecular orbital (MO)-based analysis was performed to provide chemical insight into the role of the Cl atom and the sulfinyl electronic structure in the ClSO radical.

## Experimental Methods

The experimental instrument and the theoretical methods have been previously reported.^12^ In short, a small stream of nitrogen (evaporated from liquid nitrogen) flowed through a bubbler containing Cl_2_SO (Sigma-Aldrich >99%) held in a temperature-controlled bath at 292 K (Fisherbrand, Isotemp 4100). This mixture was then introduced into a flow reactor whose pressure was monitored by a capacitance gauge (MKS 127AA-00100A) and controlled by a throttle valve (MKS type 153). The gas refreshing rate (MKS GM50A and 1179A) was slightly faster than the repetition rate of the photolysis laser (Coherent COMPex 205F, KrF). A broadband Xe plasma light source (LDLS, Energetiq EQ-99) was directed into a white cell (*R* = 140 cm, Acton Optics, 10 passes, *L*_eff_ ≈ 450 cm). The light was then projected into a two-exit grating spectrograph (Acton SpectraPro 300i), which allowed both an image-intensified CCD (Princeton Instruments PI-MAX4, 1024 × 256) and a photomultiplier tube (Hamamatsu R928) to collect transmitted light simultaneously. A long-pass filter (Semrock LP02–257RU-30 × 40) was used to separate the photolysis beam from the probed beam to reduce the background absorbance. For measurements longer than 520 nm, a long-pass filter (Thorlabs FEL0450) was placed in front of the entrance slit of the spectrometer to remove second-order diffraction. The concentrations of Cl_2_SO were in the range of (0.4–3) × 10^15^ cm^–3^ and were balanced with N_2_ for a total pressure of 40 Torr at a temperature of 292 K.

## Theoretical Methods

All equation of motion coupled cluster (EOM-CCSD) calculations were performed using the CFOUR program suite,^25^ with the exception of the unrestricted reference LR-CCSDT,^26^ which was performed using the MRCC package.^27^ All calculations made use of the ano-pVQZ basis set,^28^ which has coverage from H–Ar, as the use of atomic natural orbital (ANO) basis sets have been found to be exceptionally effective for calculating harmonic frequencies.^29^ The frozen-core approximation was applied to all calculations since the ano-pVXZ basis sets were not optimized for core correlation. The ground state was optimized using CCSD with an unrestricted Hartree–Fock (UHF) reference, while the 1^2^A′ and 2^2^A′ excited states were optimized using EOMEE-CCSD. To explore the possible effects of spin contamination, we also employed restricted Hartree–Fock (RHF) references to optimize the 1^2^A′ and 2^2^A′ excited states using the EOMEA (ClSO^+^ cation reference) and EOMIP (ClSO^–^ anion reference) approaches, respectively. The ground state was also optimized by using these two methods for calculations of the adiabatic transition energies.

Although the combination of EOM-CCSD and EOM-CCSDT provides a highly accurate treatment of dynamic correlation in the excited state, it fails to describe the behavior of the adiabatic states in a very small region about the conical intersection, yielding complex-valued energies due to the non-Hermitian character of the effective Hamiltonian.^30^ To characterize the splitting of the adiabatic states in the vicinity of the conical intersection, we turned to multireference perturbation theory, in particular XDW-CASPT2.^31^ While CASPT2 is ultimately a perturbation theory and describes dynamic correlation less accurately than coupled cluster-based methods, its extended multistate variants (*e*.*g*. XDW-CASPT2 and XMS-CASPT2) correctly describe the topology of the conical intersections. XDW-CASPT2 is especially suited for this purpose, as it interpolates between state-specific and state-averaged Fock operators as the adiabatic states approach each other and mix.

The minimum-energy conical intersection (MECI) between the 1^2^A′ and 2^2^A′ states was located via XDW-CASPT2 calculations based on a CASSCF(7,5) reference averaged over the three lowest electronic states as implemented in OpenMolcas.^32−34^ The conical intersection was located using the projected constrained optimization approach^35,36^ in which the average energy of the two states is minimized subject to the constraints^37^ that the adiabatic energy difference is zero and a dummy constraint that prevents motion along the analytically computed^38^ derivative coupling vector. An imaginary shift of 0.05 eV was applied for all calculations to avoid intruder states. The weighting factor used for the dynamically weighted Fock matrix was as described in ref (39), corresponding to the OpenMolcas keywords of DWType = 3 and DWMS = 1.0. The conical intersection was characterized in the branching plane by displaced geometries in the plane defined by the ***x*** and ***y*** vectors, which span the same plane as the ***g*** and ***h*** vectors and are related to them by rotation.^37^ The displacements formed a grid in polar coordinates with a spacing of 0.05 Å in ***R*** and 20° in **θ**. Parallel computations were aided by the use of GNU parallel.^40^

The absolute cross section as a function of wavelength was simulated in a rough approximation neglecting coupling between the 1^2^A′ and 2^2^A′ states as the sum of two independent spectral contributions. Franck–Condon factors for the 2^2^A′ ← X^2^A″ transition at 0 K including Duschinsky rotation effects were calculated using the ezFCF package,^41^ using vibrational frequencies and 0–0 transition energies taken from the EOMEE-CCSD/ano-pVQZ calculations. The absolute contribution to the cross section is given by
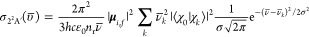
where σ is an empirical broadening parameter chosen to correspond to a full width at half-maximum of 100 cm^–1^. The 1^2^A′ contribution to the spectrum was estimated using a multidimensional extension of the reflection principle^42^ and is given by
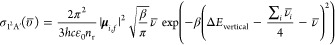
where β^–1^ is the norm of the gradient of the upper state in cm^–1^ (see ref (42)), and the ν̅_*i*_ are the ground-state vibrational frequencies (expressed in cm^–1^). EOMEE-CCSD was used for the vertical excitation energy, excited-state gradient, and transition dipole moment. The ground-state CCSD vibrational frequencies were used for the ν̅_*i*_.

Orbitals were visualized with the IBOView package,^43^ while vibrational arrow diagrams were generated with the PyVibMS plugin^44^ to pymol.^45^

## Results

### Experimental ClSO A-Band Spectrum

**Figure 1:**
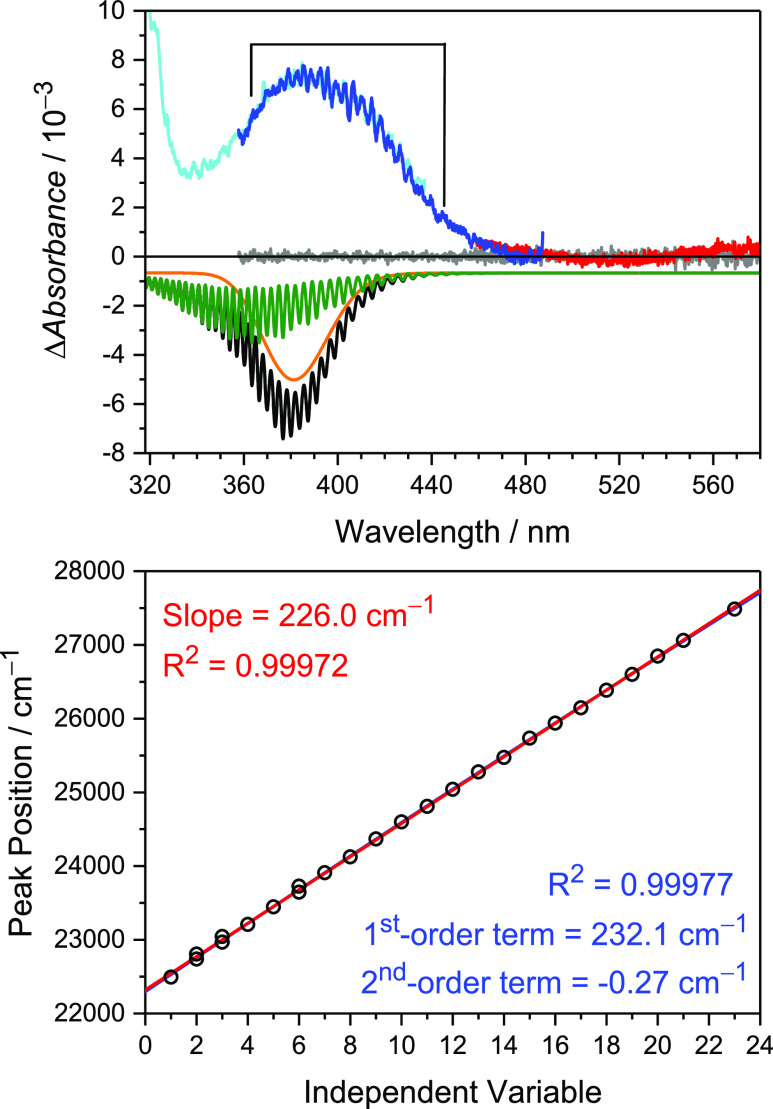
(Upper panel) Recorded spectra of the Cl_2_SO/N_2_/248 nm system at 100 μs after pulsed laser (exposure time = 117.5 μs, averaged for 12,288 shots) at 40 Torr and 292 K. Cyan, blue, and red lines represent spectra at different grating angles (center wavelengths of 370, 420, and 520 nm), while the gray lines show the background noise without Cl_2_SO. The black simulated spectra downward is the sum of contributions from both the 1^2^A′ (orange) and 2^2^A′ (olive, stick spectra convoluted with a Gaussian function with fwhm = 100 cm^–1^) states. The comparison between experimental and theoretical absolute cross sections is shown in Figure S5. (Lower panel) Linear and quadratic fits to the observed positions of the vibrational peaks within the marked region.

Figure 1 shows representative spectra of the Cl_2_SO/N_2_ photolysis system at 40 Torr, recorded at a delay time of 100 μs. We have previously reported a strong absorption below 320 nm, which has a maximum at 303 nm, and assigned it to the 1^2^A″ ← X^2^A″ transition, the B band of the ClSO radical.^12^

We found another weak absorption with a maximum peak position near 385 nm, exhibiting a series of vibronic bands. The apparent threshold of this weak absorption feature is around 480 nm. The slight variations in baseline above 500 nm show the uncertainty in baseline under high [Cl_2_SO]. The precursor may also have a small absorption (Figure S1). A fit to the partially resolved vibrational progression observed in the experiment yielded an average spacing of 226 cm^–1^. The progression was linear over the range observed; as shown in Figure 1, fitting to a quadratic function was not a statistically significant improvement.

The weak absorption band centered near 385 nm has a decay behavior identical to the absorption near 303 nm to within the experimental uncertainty (Figure S2). The intensity was found to be proportional to the concentration of Cl_2_SO in the reactor (Figures S3 and S4) when [Cl_2_SO] < 1.3 × 10^15^ cm^–3^. The concentrations of Cl_2_SO used in this experiment were quite high ([Cl_2_SO] ≈ 3 × 10^15^ cm^–3^) to ensure a large enough transient signal. However, this high concentration also resulted in a strong absorption of the photolysis laser (σ(248 nm) = 7.05 × 10^–18^ cm^2^,^46^*L* = 45 cm, *T* = 37.7%), leading to inhomogeneous radical formation throughout the flow reactor along the excimer path (Figure S4). As a result of these conditions, we did not attempt a quantitative kinetic analysis of the time-dependent data to obtain the rate constants. Based on the concentration dependence and temporal behavior at distinct wavelengths, we tentatively assigned the origin of the weak band to ClSO radicals.

We calibrated the absolute cross section of the weak band near 385 nm from the absorbance of the 1^2^A″ ← X^2^A″ transition in the 310–320 nm range, which has a peak cross section, σ(303 nm) = (2.0 ± 0.5) × 10^–18^ cm^2^.^12^Figure S5 demonstrates that the weak band has a maximum absorption cross section σ(385 nm) = (7.6 ± 1.9) × 10^–20^ cm^2^ near 385 nm (1 standard deviation).

### EOM-CCSD Calculations

Table 1 summarizes the results of coupled cluster point calculations for the X^2^A″ ground state and the two lowest lying ^2^A′ excited states. The 1^2^A′ state arises from the α SOMO–LUMO transition, and the 2^2^A′ state arises from the β SOMO-1 to SOMO transition. All three states have bound minima. The adiabatic transition energy of the 2^2^A′ state was found to be 20,463 cm^–1^, higher than that of the 1^2^A′ state (16,122 cm^–1^); thus, there was no change in the energy ordering of the ^2^A′ excited states. The minimum energy of the 2^2^A′ state is predicted to be higher than the bond dissociation energy *D*_0_(Cl–SO) = 19,048 cm^–1^ using the HEAT-345(Q) method^47−50^ (see Table S1), while the minimum of the 1^2^A′ state is predicted to be below *D*_0_(Cl–SO). The observed absorption band lies well above dissociation.

**Table 1:** Summary of the Calculations of ClSO for Geometries, Harmonic Frequencies, Adiabatic Transition Energies Δ*E* Relative to the Ground State, and the 0–0 Transition Energies

SCF reference		UHF/EOMEE			RHF/EOMIP (anion)		RHF/EOMEA (cation)	
geometry-optimized		X^2^A″	2^2^A′	1^2^A′	X^2^A″	2^2^A′	X^2^A″	1^2^A′
geometry^a^	*r*(S–O)/Å	1.461	1.672	1.459	1.464	1.643	1.453	1.460
	*r*(Cl–S)/Å	2.046	2.013	2.276	2.034	2.012	2.035	2.259
	∠(ClSO)/°	109.25	91.50	152.69	109.42	90.19	109.57	149.78
frequency^a^/cm^–1^	ClSO bending	316.3	226.5	214.3	320.6	221.0	323.1	232.9
	ClS stretching	515.5	550.7	333.3	541.8	544.6	533.8	345.1
	SO stretching	1199.5	735.4	1197.7	1190.3	802.8	1248.3	1195.4
Δ*E*/cm^–1^	CCSD/ano-pVQZ	0	20,697	18,517	0	21,027	0	17,558
			(20,742)^b^	(16,759)^b^		(20,463)^c^		(16,122)^c^
0–0 transition energy/cm^–1^ [nm]			20,482	16,616		20,221		15,956
			[488.2]	[601.8]		[494.5]		[626.7]

a Optimization and frequency at the CCSD/ano-pVQZ level.

b Correction for the triples by Δ*E*_HLC_ = CCSDT/ano-pVDZ – CCSD/ano-pVDZ similar to ref (47).

c At the CCSDT/ano-pVQZ level.

The 1^2^A′ excited state is characterized by a large ClSO angle and a longer ClS bond length than those of the X^2^A″ state. The 2^2^A′ excited state has an almost 90° ClSO angle and a longer SO bond length and is closer to the geometry of the ground state. Both ^2^A′ excited states have lower bending frequencies (214 and 227 cm^–1^) than the ground state (316 cm^–1^). The 1^2^A′ state has a lower ClS stretching frequency (333 cm^–1^) than the ground state, while 2^2^A′ has a lower SO stretching frequency (735 cm^–1^); both results are consistent with the respective bond length changes. Further calculations utilizing closed-shell references produced results that were similar to those of the EOMEE calculations, suggesting minimal spin contamination effects.

### Assignment of the 385 nm Spectrum

Both low-lying A′ excited states are predicted to have similar vertical excitation energies consistent with the observed band at 385 nm.^12^ The transition to the 1^2^A′ state is dissociative, while the transition to 2^2^A′ is bound. The observed cross section is within a factor of 2 of the theoretical absolute cross-section maximum, σ_the_(377 nm) = 1.8 × 10^–19^ cm^2^. The cross section relative to that of the strong Bband is consistent with the oscillator strengths that we previously predicted. We found *f*(1^2^A′) = 3.89 × 10^–4^, *f*(2^2^A′) = 2.88 × 10^–4^, and *f*(1^2^A″) = 1.22 × 10^–2^ for transition to the higher excited states.^12^ The ratio of the predicted oscillator strengths is (*f*(1^2^A″)/(*f*(1^2^A′) + *f*(2^2^A′))= 18.0, consistent with the ratio of the observed absolute cross sections at the peaks, σ(305 nm)/σ(385 nm) = 2.0 × 10^–18^ cm^2^/7.6 × 10^–20^ cm^2^ = 26.3.

**Figure 2:**
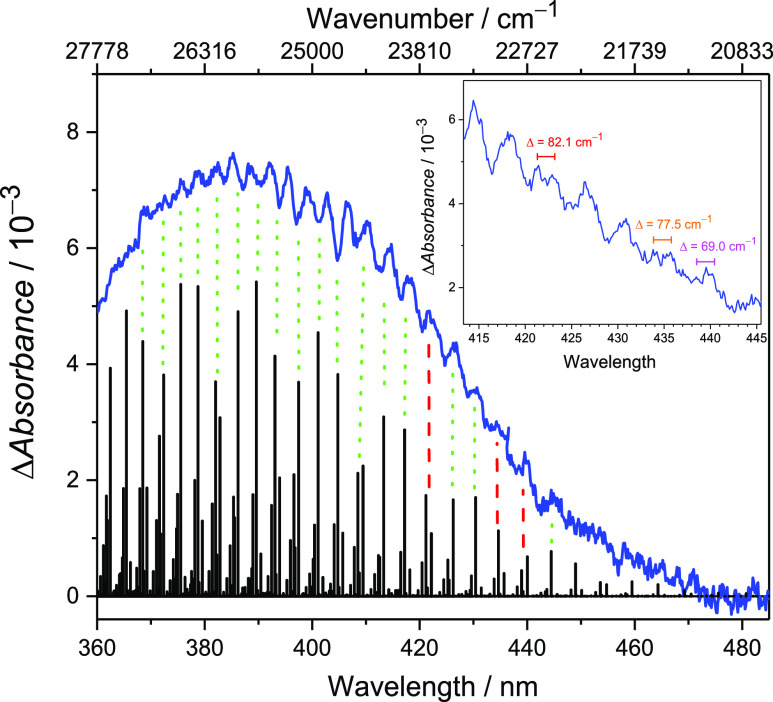
Closer look at the structure of the weak absorption band in the 360–480 nm range. A few doublet peaks were observed, yielding an average spacing of 76 cm^–1^. The stick spectrum displays the intensities predicted for excitation to the 2^2^A′ state, incorporating Franck–Condon factors and thermal population effects at 0 K. The energy of the computed transition origin has been shifted to red by 870 cm^–1^ for alignment with the vibronic progression of the experimental spectrum (green dotted lines), while the red dashed lines indicate predicted doublets. Inset is a magnification of the spectrum between 415 and 445 nm.

The bending frequencies predicted for both ^2^A′ states agree well with the experimentally observed vibrational progression of 266 cm^–1^ (see Figure 1). In addition, Figure 2 shows doublet peaks at 422, 435, and 439 nm, which may be attributed to the difference between the SO stretching frequency and three times the bending frequency of the 2^2^A′ state. For instance, the doublet peak at 435 nm could correspond to the (4,2,0) ← (0,0,0) and (3,5,0) ← (0,0,0) transitions, leading to an estimated frequency for the SO stretching mode of 755 cm^–1^. From the calculated Franck–Condon contour, we extrapolated the origin to be roughly near 490 nm (20,400 cm^–1^), which is consistent with the 0–0 transition energy of the 2^2^A′ state shown in Table 1.

The observed vibrational structure disappears on the blue side, while the simulated spectrum exhibits a clear vibrational progression of the 2^2^A′ state across the whole band. The peak broadening on the blue side might be due to congestion and anharmonicity or decreases in the lifetime of the 2^2^A′ excited state caused by internal conversion (*vide infra*, see ″Influence of the Conical Intersection″).

A few peaks within the 320–360 nm range (Figure S6) were also observed, which have a small spacing of approximately 367 cm^–1^ and a large spacing of 1060 cm^–1^. These energy differences are close to the calculated ClS stretching and SO stretching frequencies of the 1^2^A′ state and may warrant further theoretical investigation.

As a first-order approximation, we modeled the spectrum with the EOMEE-CCSD/ano-pVQZ results, assuming that the spectrum was the incoherent sum of the 1^2^A′ ← X^2^A″ and 2^2^A′ ← X^2^A″ transitions weighted by the computed oscillator strengths (see Figure 1). We treated the bound-to-bound transition to the 2^2^A′ state as two independent displaced harmonic oscillator models based on the minimum-energy geometries of the X^2^A″ and 2^2^A′ states adopted from the EOMEE-CCSD/ano-pVQZ calculations including Duschinsky rotation at 0 K (Figure S7). We simulated the 1^2^A′ ← X^2^A″ transition using a multidimensional extension of the reflection principle. The two states made roughly equal contributions.

### Prediction of a Conical Intersection between the 1^2^A′ and 2^2^A′ States

Our previous calculations found that the vertical excitation energies of the two ^2^A′ states were very close in energy. This result suggested that a conical intersection between the 1^2^A′ and 2^2^A′ states might exist near the ground-state geometry. To investigate this phenomenon, we generated a cut of the bending potential energy curve by linearly connecting the geometries of X^2^A″, 1^2^A′, and 2^2^A′ states (Figure S8). We employed the EOMIP and EOMEA approaches to treat 1^2^A′ and 2^2^A′ independently (Figure S9). In addition, the EOMEE/ano-pVTZ calculation failed to converge at the upper state (Figure S10).

To locate the conical intersection, we explored the lowest three potential energy surfaces (PES) of the ClSO radical using XDW-CASPT2^31,39^ with a seven-electrons in five-orbitals CASSCF reference. The active orbitals in the ground-state equilibrium geometry are pictured in Figure 5. The resulting equilibrium geometries and relative energies are summarized in Table 2.

**Table 2:** Summary of the Geometries and Energy Difference Relative to the Ground State, Δ*E*, Calculated at the XDW-CASPT2(7,5) Level

geometry-optimized		X^2^A″	1^2^A′	2^2^A′	MECI
geometry	*r*(S–O)/Å	1.446	1.480	1.688	1.593
	*r*(Cl–S)/Å	2.007	2.259	1.997	2.010
	∠(ClSO)/°	111.8	156.1	93.0	119.2
	**x** (branching space)/Å	–0.0283	–0.4444	0.2473	0
	**y** (branching space)/Å	–0.0549	0.1769	–0.1508	0
	**z** (seam space)/Å	0.1611	–0.4138	0.3517	0
Δ*E*/cm^–1^		0	14,431	23,198	26,414

**Figure 3:**
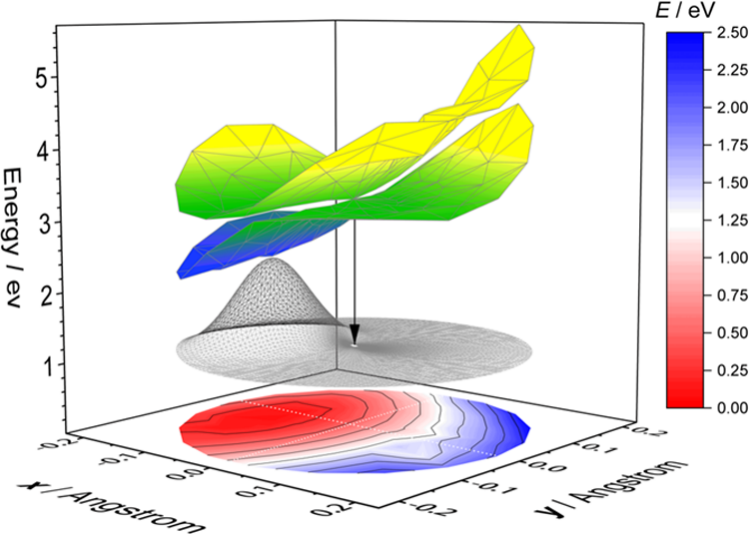
Adiabatic potential energy surface of the ground state and two excited ^2^A′ states of the ClSO radical in the 2D branching space (*x*, *y*; see Figure 4) computed at the XDW-CASPT2(7,5)-3SA level near the MECI geometry (*x* = 0, *y* = 0). The meshed line shows the profile of the ground-state vibrational wave function. The black arrow shows the vertical transition from the MECI to the ground-state PES (which is flattened for clarity; the color bar to the right gives the energy scale of the ground-state PES contours).

We found a sloped-type conical intersection of the 1^2^A′ and 2^2^A′ states, as depicted in Figure 3, with a minimum-energy conical intersection at 26,414 cm^–1^, close to the peak of the absorption. The geometries of the MECI and ground state are similar, with the MECI SO bond being slightly longer. Indeed, we found that the change between X^2^A″ and MECI in the branching space is small (0.06 Å, Table 2) with a larger change in seam space, indicating that the two ^2^A′ excited-state PES do cross near the Franck–Condon region, although the MECI is slightly far away from the ground-state geometry.

**Figure 4:**
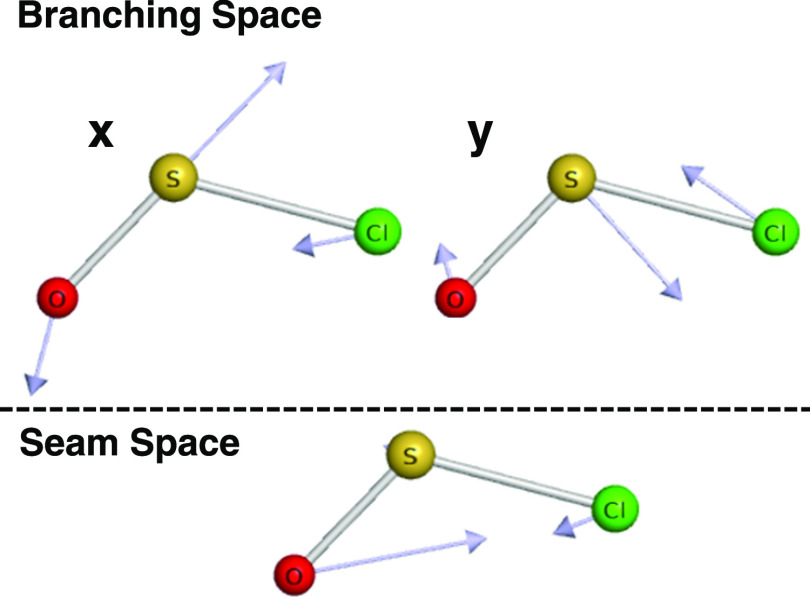
Corresponding vibrational motions of the seam space (the set of geometries where 1^2^A′ and 2^2^A′ states are degenerate) and the branching space (the complement of the seam space) near the MECI geometry.

The corresponding motions of **x** and **y** vectors^37^ in the branching space as well as the motions in seam space are also illustrated in Figure 4. Motion along the **x** vector (large contribution from the antisymmetric stretch) on the lower surface lowers the energy, presumably leading to the 1A′ well (contracting the SO bond and elongating the SCl bond). Motion along the **y** vector mainly consisted of symmetric stretching.

The bending mode is the primary motion along the seam space (Figure 4), *i*.*e*., the degeneracy of the conical intersection is preserved as the molecule bends. The bending motion thus has the weakest vibronic coupling among the three vibrational modes. As a result, we expect the lifetimes of the bending vibronic excited states to be longer than other modes, consistent with the observation of the bending motion as the most prominent structural feature in the experimental spectrum.

## Discussion

### Influence of the Conical Intersection

Our current time-independent, low-resolution absorption spectrum does not provide more information about the interactions between both A′ states near the conical intersection. We attributed the observed structure to the 2^2^A′ ← X^2^A″ state, but both the 1^2^A′ and 2^2^A′ states contribute to the observed band. While we simulated the spectrum as an incoherent excitation of the two adiabatic states, the true excitation process produces a superposition of the two states and the coefficients of each state vary with energy, in part due to changing vibronic coupling across the band. Additionally, the excited wave packet would undergo nonadiabatic dynamics on the coupled 1^2^A′ and 2^2^A′ states, especially near the conical intersection, resulting in a shorter lifetime and account for the loss of the vibrational structure toward shorter wavelengths.

The predominant Franck–Condon active mode in the vibrational progression of the observed spectrum is the ClSO bending mode. This is consistent with the prediction of the bending mode as the motion along the seam space. However, broadening at shorter wavelengths and observation of the bending vibrational progression could result from other effects, as discussed above, and therefore only provide circumstantial evidence for the presence of a conical intersection.

The fate of the excited state of the ClSO radical is unclear. The molecules excited to the 2^2^A′ state could relax to the lower 1^2^A′ state via the conical intersection along the **x** coordinate; subsequent collisions could further relax the molecule below the dissociation limit into the bound 1^2^A′ well, at which point the molecule could reach the ground state by internal conversion, collisional quenching, or fluorescence.

To quantitatively predict the effects of the conical intersection on the observed spectrum is challenging and goes beyond the scope of this study. Use of a single-shot vibronic coupling model based on EOM-CCSD in the spirit of Ichino et al.^51^ is complicated by the lack of a single, closed-shell, noninteracting reference state. Furthermore, the two electronic states share the same irreducible representation, leading to orbital mixing at the SCF level resulting in diabats that hold little chemical significance.

### Molecular Orbital Diagram of the ClSO Radical

**Figure 5:**
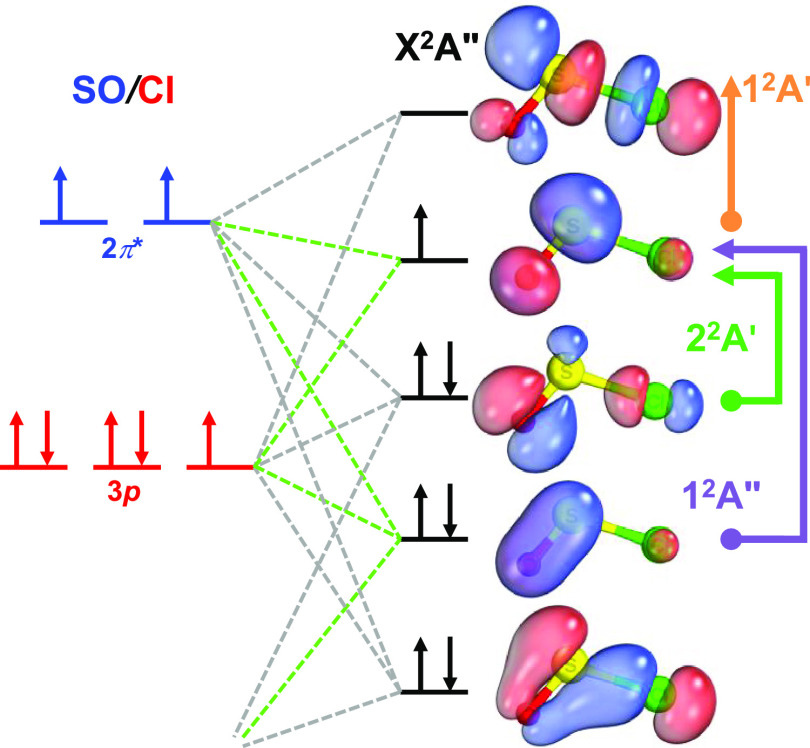
Schematic MO diagram showing the active space orbitals of the ClSO radical (top view) used in the XDW-CASPT2 calculation. Vertical and horizontal arrows indicate transitions to excited states for the α and β electrons. The green lines connect orbitals with an out-of-plane orientation.

Figure 5 summarizes the valence electronic structure of the ClSO radical. To construct this MO diagram, we used the ionization energies of the SO molecule (10.3 eV)^52^ and Cl atom (13.0 eV)^53^ to establish the relative energy positions.

Figure S11 depicts the nine molecular orbitals resulting from the combination of 6σ, 2π, 2π*, and 7σ orbitals of SO, along with the 3p orbitals of the Cl atom under *C*_s_ symmetry. These orbitals can be broadly classified as bonding, antibonding, and nonbonding. The term nonbonding here is used loosely, referring to orbitals that exhibit both bonding and antibonding characteristics simultaneously along distinct chemical bonds.

For instance, the second occupied molecular orbital (SOMO-1) is a combination of an in-plane π* orbital along the SO bond and a σ orbital along the ClS bond. This can be interpreted as the SO moiety being stabilized by donating electrons from a π* orbital into the 3*p* orbital of the Cl atom. We may expect that the reactivity of the ClSO radical does not change significantly with respect to the SO radical, supported by the nearly identical SO bond length compared to free SO radicals (*r*_SO_ = 1.48 Å).^54^ Since the ClSO + Cl → Cl_2_SO reaction has been reported,^12^ we expect that the association reaction of SO + Cl → ClSO also occurs.

This MO analysis of the ClSO radical provides insights into the electronic structure of other sulfinyl radicals as the ionization energy of the Cl atom 3*p* orbital is similar to the ionization energies of H, C, N, and O atoms.^55^ Typically, a radical species possesses seven electrons, occupying three perpendicular orbitals similar to those of the Cl atom. Additionally, we predict that transitions from the SOMO-1 to the SOMO orbital are likely to occur in other sulfinyl radicals, resembling the 2^2^A′ ← X^2^A″ transition observed in ClSO. Furthermore, a strong π*_SO_ ← π_SO_ transition will also be present, similar to the ClSO 1^2^A″ ← X^2^A″ transition. Indeed, the literature has reported the presence of these two distinct transitions in certain sulfinyl radicals, as summarized in Table 3.

**Table 3:** Summary of the UV–Vis Absorption Band Positions of Distinct Sulfinyl Radicals (RSO)

unit/nm	π*_SO_ ← π_SO_	SOMO ← SOMO-1	sample phase
SO	190–240		gas^56^
H–SO		520–960^a^	gas^57^
HO–SO	∼270	300–500	Ne-matrix
H_3_C–SO	260–300	450–635	Ar-matrix^20^
F_3_C–SO	250–300	490–610	Ar-matrix^23^
C_6_H_5_–SO	260–350	410–470	Ar-matrix^22^
	∼300	∼450	solution(C_6_H_12_)^58^
H_2_CC(H)–SO	240–310	350–490	N_2_-matrix
Cl–SO	260–320	350–460	gas

a Chemiluminescence emission spectrum.

Peroxyl radicals (RO_2_) are approximately isoelectronic molecules of sulfinyl radicals. Experiments have shown that two transitions within the near-IR and UV–vis regions are commonly observed in peroxyl radicals. Weisman and Head-Gordon^59^ have successfully explained the observed trend in band positions using a MO picture.

Building upon similar concepts, we have concluded that the π*_SO_ ← π_SO_ transition in sulfinyl radicals occurs at a similar band position due to the minimal mixing of the orbital character from the substitution group. Conversely, the band positions of SOMO ← SOMO-1 transitions cover a wide range of wavelengths because the energy of the SOMO-1 orbital depends on the characteristics of the substitution groups.

It is noteworthy that the chemiluminescence spectrum of HSO has been detected^57^ in the visible range for the SOMO ← SOMO-1 transition,^60^ which suggests that the ClSO radical might fluoresce after excitation to the A band and could work as a better tool to study the chemical reactivity and kinetics.

## Conclusions

In this work, we present the UV–vis absorption spectrum of the ClSO radical in the gas phase, in the 320–500 nm range at 40 Torr and 292 K, denoted here as the A band. We observed a new, weak band having a partially resolved vibrational progression with an average spacing of 226 cm^–1^. We determined the peak cross section by calibrating it with the cross section of the much stronger B band at 303 nm.

We assigned the band as excitation to the continuum of the 1^2^A′ excited state and the bound 2^2^A′ excited state. Using the EOM-CCSD/ano-pVQZ method, we calculated the properties of the two doublet excited states in this region, both with A′ symmetry in the *C*_s_ point group. Both states could contribute to the absorption intensity since they have similar vertical excitation oscillator strengths. The 1^2^A′ excited state had a minimum below the dissociation limit and possessed a larger bond angle and longer ClS bond length, while the 2^2^A′ excited state lying above the dissociation limit exhibited a nearly 90° bond angle and a longer SO bond length. We simulated the spectrum as a sum of the bound–free 1^2^A′ ← X^2^A″ transition and the bound–bound 2^2^A′ ← X^2^A″ transition. By comparing the calculated harmonic frequency and predicted 0–0 transition energy, we assigned the observed vibronic progression to the bending mode of the 2^2^A′ ← X^2^A″ transition.

We conducted XDW-CASPT2 calculations and identified a conical intersection between the 1^2^A′ and 2^2^A′ excited states. The geometry of the minimum-energy conical intersection was found to be similar to the ground-state geometry, indicating that the conical intersection was near the Franck–Condon region of the A band transition, suggesting that the two ^2^A′ excited states will mix due to strong vibronic coupling; a short lifetime of the 2^2^A′ state is expected and could account for the unresolved vibrational progression on the blue side of the band.

The observed spectrum provides only indirect evidence for the presence of this conical intersection. A complete theoretical understanding of the spectroscopic effects of this phenomenon will require accurate descriptions of the potential energy surfaces, vibronic couplings, and dynamical effects. Experimentally, the existence of the conical intersection could be demonstrated by high-resolution spectra, fluorescence spectra, and probes of possible photodissociation dynamics (*e.g.*, molecular-beam photofragment-translational spectroscopy or stimulated Raman spectroscopy).

Finally, we constructed a simplified MO diagram to illustrate our understanding of the electronic structure of the ClSO radical, which also highlights similarities to those of other sulfinyl radicals. This diagram serves as a useful tool in understanding the trends of the observed band positions, considering the effects of substitution groups on the orbital characteristics. In general, species containing the sulfinyl group can be anticipated to display strong absorption around 300 nm, while weaker bands with distinct features are expected within the range 400–600 nm.

## Supporting Information Available

Experimental details and analyses; experimental and theoretical absorption cross sections available via the Caltech Research Data Repository (PDF)
