# Supplementary material for: A-Band Absorption Spectrum of the ClSO Radical: Electronic Structure of the Sulfinyl Group
Source: J Phys Chem A. 2023 Sep 29;127(40):8374–82. doi: 10.1021/acs.jpca.3c04977 (PMC10577680; doi:10.1021/acs.jpca.3c04977)
Supplement: Supplementary file 1 — jp3c04977_si_001.pdf [file jp3c04977_si_001.pdf]

A-Band Absorption Spectrum of the ClSO Radical: Electronic Structure of the Sulfinyl Group

Wen Chao,[‡,*] Gregory H. Jones,[‡] Mitchio Okumura,[‡,*] Carl J. Percival,[†] Frank A. F. Winiberg[†,*]

[‡] Division of Chemistry and Chemical Engineering, California Institute of Technology, 1200 E California Blvd, Pasadena, CA 91128 United States

[†] Jet Propulsion Laboratory, California Institute of Technology, 4800 Oak Grove Drive, Pasadena, CA 91109-8099, United States

*Email: fred.a.winiberg@jpl.nasa.gov

*Email: mo@caltech.edu

*Email: wchao@caltech.edu

Table of Contents

Absorption Spectrum of the Cl_2SO Sample (Fig S1) S2

Comparison of the Temporal Profiles (Fig S2) S3

Concentration Dependence of the Weak Band (Fig S3−S4) S4

Absorption Cross Section of ClSO (Fig S5) S5

Vibrational Progression Analyses (Fig S6) S5

Simulated Spectrum of displaced Harmonic Oscillator Models (Fig S7) S6

Bending Potential Energy Curves (Fig S8−S10) S7

Detailed Contributions of HEAT Methods (Table S1) S8

Molecular Orbital Diagram of ClSO Radical (Fig S11) S9

References S10

Absorption Spectrum of the Cl_2SO Sample

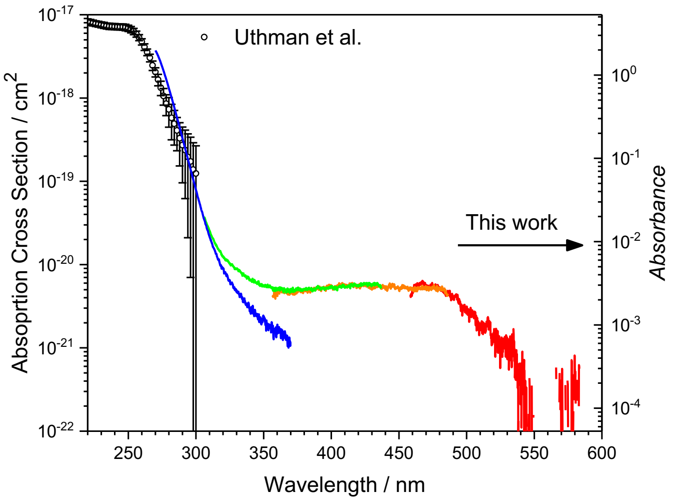

Figure S1. Absorption spectrum of the Cl_2SO sample used in this study without further purifications. The distinct spectral lines, represented by various colors, correspond to different centers of wavelength. The concentration of Cl_2SO was adjusted to be 3.1×10^{15} molecules cm^{-3}, the same as the experimental conditions shown in Figure 1. The black circles indicate the absorption cross section from Ref 1.

Comparison of the Temporal Profiles

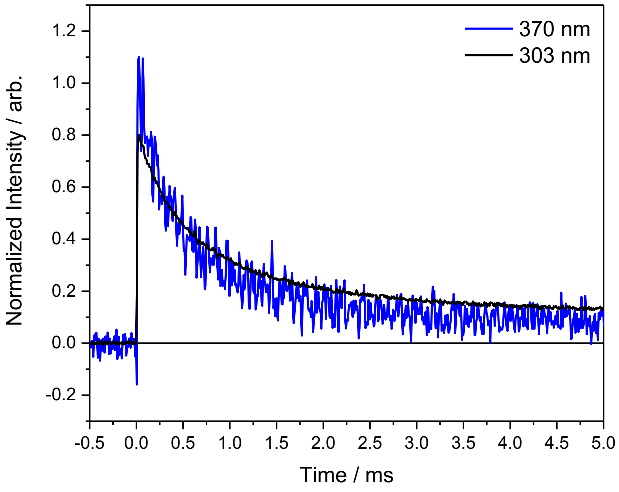

Figure. S2. Temporal profiles of the Cl_2SO/N_2/248 nm system, monitored near 303 nm (black, $1^2A'' \leftarrow X^2A''$) and 370 nm (blue, $2^2A' \leftarrow X\ ^2A''$) under a pressure of 40 Torr. The observed temporal profiles are roughly consistent with each other, although the decay rate at 303 nm is slightly slower. Given the high concentration of Cl_2SO ($[Cl_2SO] = 7.5\times10^{14}$ cm^{-3}), the products from the secondary chemistry, which could generate species with the SO group and exhibit strong absorption near 300 nm, might be observed.

Concentration Dependence of the Weak Band

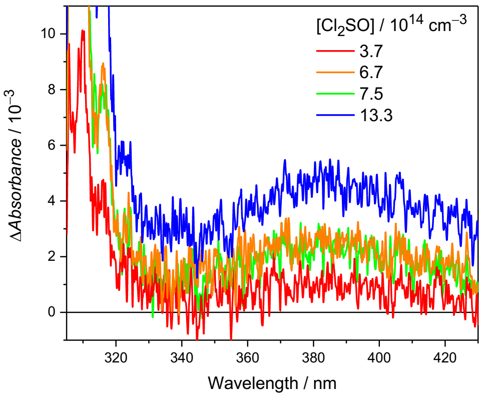

Figure. S3. Recorded spectrum of the weak absorption band (Center Wavelength = 370 nm) under distinct [Cl_2SO] for 2048 shots average.

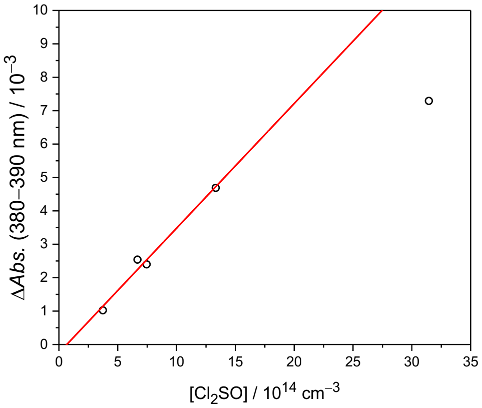

Figure. S4. Plot of observed Δ*Abs*. (380−390 nm average) versus [Cl_2SO], including the data from Figure 1 and Figure S3. At the highest [Cl_2SO] concentrations, the observed absorption deviates from the linear region, showing the effect of absorption from the $C_{12}SO$ sample affecting the photolysis laser.

Absorption Cross Section of ClSO

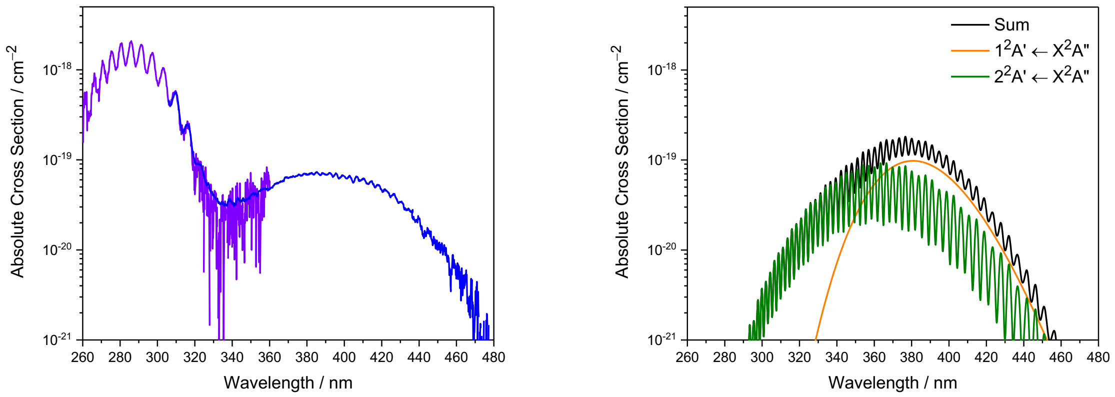

Figure. S5. (Left) Comparison of absorption cross sections for the 1^2A" ← X^2A" transition (black, Ref 2) and the 2^2A'←X ^{2}A" transition (blue) of ClSO radical. (Right) The calculated absolute cross section of both the 1^2A" ← X^2A" and the 2^2A'←X ^{2}A" transitions.

Vibrational Progression Analyses

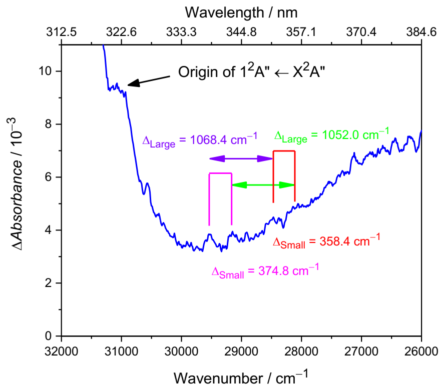

Figure. S6. A closer look of the small structures in the valley region within the 312.5–385 nm. The origin of the 1^2A" ← X^2A" transition is also indicated.

Simulated Spectrum of Displaced Harmonic Oscillator Models

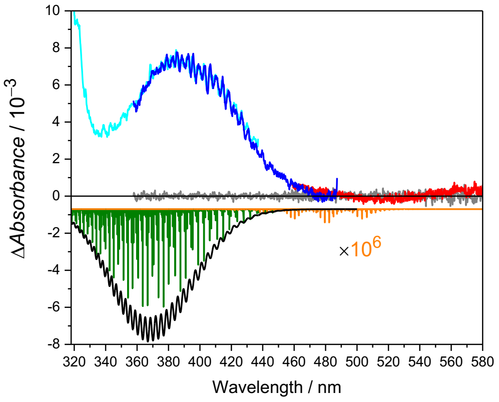

Figure. S7. The recorded spectrum of the $Cl_2SO/N_2/248$ nm system at 100 μs after pulsed laser (exposure time = 117.5 μs, averaged for 12288 shots) under 40 Torr. Cyan, blue, and red lines represent spectra at different grating angles (center wavelengths of 370 nm, 420 nm, and 520 nm), while the gray lines show the background noise without Cl_2SO. The downward stick spectra are the transitions to the $1^2A'$ (orange, $\times10^6$) and $2^2A'$ (olive) states predicted by the EOMEE-CCSD/ano-PVQZ calculations. Note that the Frank-Condon factors of the $1^2A'$ state is six orders of magnitude smaller than the $2^2A'$ state. The black line is the simulated spectrum to both states (stick spectra convoluted with a Gaussian function with FWHM = 150 cm^{-1}).

Bending Potential Energy Curves

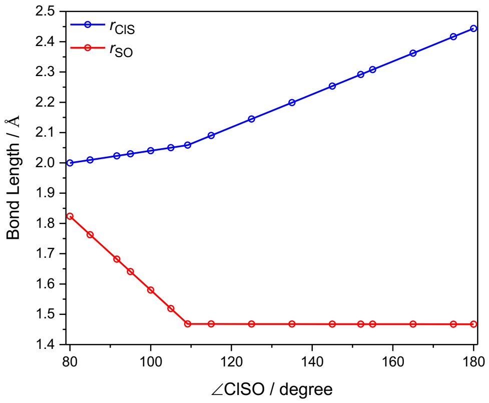

Figure. S8. Changes in ClS, r_{ClS}, and SO, r_{SO}, bond length in the scan of the bending potential energy curves, as represented in Figure S8-S9. The coordinates were generated by linearly connecting the energy minimum geometries of the $2^2A'$, X^2A'' and $1^2A'$ excited states in the order from small angle to large angle.

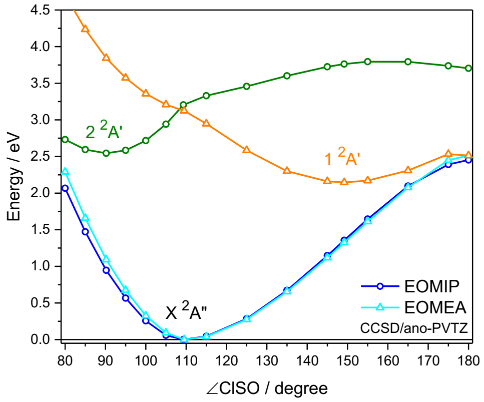

Figure. S9. Bending potential energy curves calculated at the EOMEA-CCSD/ano-pVTZ level for the $1^2A'$ excited state (orange) and at the EOMIP-CCSD/ano-pVTZ level for the $2^2A'$ excited state (olive). Associated geometric parameters are given in Figure S7. A cross between these two curves near the minimum X^2A'' geometry suggests the existence of a conical intersection.

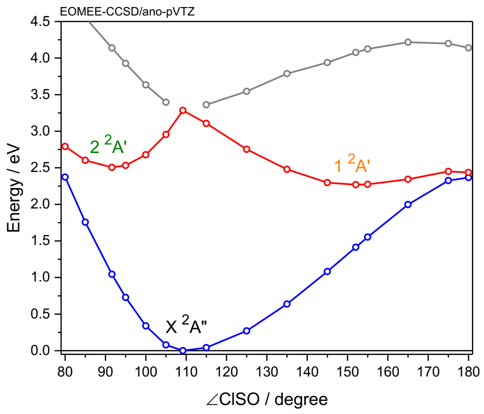

Figure. S10. Bending potential energy curves calculated at the EOMEE-CCSD/ano-pVTZ level for the 1^2A' and 2^2A' excited states. Associated geometric parameters are given in Figure S7. At the minimum X^2A" geometry, the excitation energy for the second A' state failed to converge.

Detailed Contributions of HEAT Method

Table S1. Individual contributions to the HEAT total energies (in Hartree) and the standard enthalpy change at 0 K.

Species	E^{∞}_{HF}	$\Delta E^{\infty}_{CCSD(T)}$	ΔE_{CCSDT}	ΔE_{CCSDTQ}	ΔE_{REL}	ΔE_{DBOC}	$\Delta E_{SO}{}^{a}$	ΔE_{ZPE}	Total
Cl	-459.489895	-0.665245	-0.000767	-0.000161	-1.404007	0.005940	-0.001338	0.000000	-461.555473
ClSO	-931.946359	-1.683349	-0.000890	-0.001801	-2.533727	0.013978	0.000000	0.004539	-936.147609
SO	-472.421430	-0.962796	-0.000481	-0.001299	-1.129986	0.008022	0.000000	0.002626	-474.505345

Reaction	ΔH°(0 K, HEAT) / kJ mol^{-1}
ClSO → Cl + SO	227.869

[a] Values adopted from the CCCBDB database, II.C.2 Electronic Spin Splitting Corrections.

Molecular Orbital Diagram of ClSO Radical

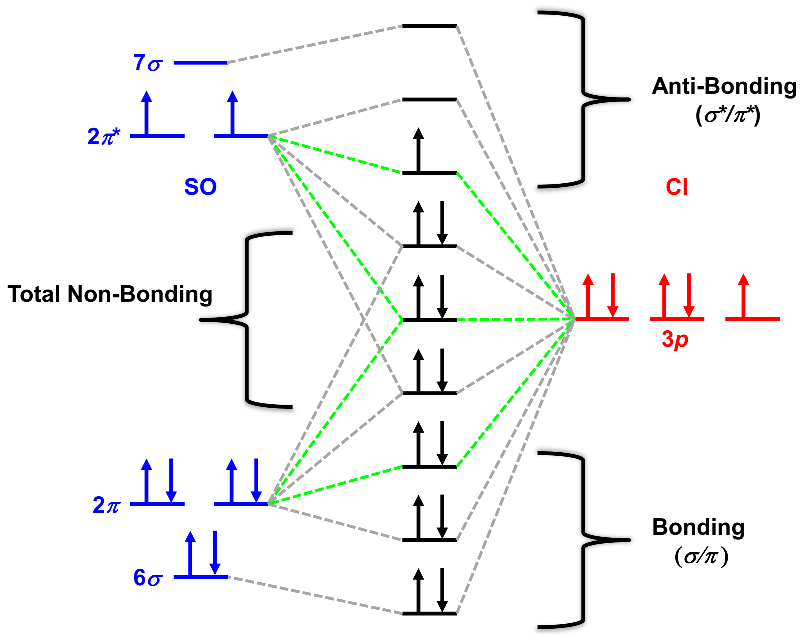

Figure. S11. The molecular orbital diagram of ClSO radical, considering the valence orbitals of SO molecules and the 3*p* orbital of Cl atoms. The green lines indicate the interaction of out-of-plane (A") orbitals. Overall, the total 9 molecular orbitals are classified as bonding, total non-bonding and anti-bonding.

References

(1) Uthman, A. P.; Demlein, P. J.; Allston, T. D.; Withiam, M. C.; McClements, M. J.; Takacs, G. A. Photoabsorption Spectra of Gaseous Methyl Bromide, Ethylene Dibromide, Nitrosyl Bromide, Thionyl Chloride, and Sulfuryl Chloride. *J. Phys. Chem.* **1978**, *82* (20), 2252–2257. https://doi.org/10.1021/j100509a021.

(2) Chao, W.; Jones, G. H.; Okumura, M.; Percival, C. J.; Winiberg, F. A. F. Spectroscopic and Kinetic Studies of the ClSO Radical from Cl_2SO Photolysis. *J. Am. Chem. Soc.* **2022**, *144* (44), 20323–20331. https://doi.org/10.1021/jacs.2c07912.
